# Development and validation of a tool to assess knowledge of healthy lifestyles in early grade school children

**DOI:** 10.1186/s13104-018-3332-7

**Published:** 2018-04-03

**Authors:** Salim R. Surani, Sean Hesselbacher, Zoya Surani, Moinuddin Mokhasi, Saherish S. Surani, Jose Guardiola, Lauren Quisenberry, Sara S. Surani

**Affiliations:** 1 Texas A&M University, Corpus Christi, TX USA; 2 Hampton VA Medical Center, Hampton, VA USA; 3 Eastern Virginia Medical School, Norfolk, VA USA; 4 Veterans Memorial School, Corpus Christi, TX USA; 5 Driscoll Children Hospital, Corpus Christi, TX USA; 6 Department of Mathematics and Statistics, Texas A&M University Corpus Christi, Corpus Christi, TX USA; 7 University of Texas, El Paso, TX USA; 8 Harvard University, Boston, MA USA; 9 701 Ayers Street, Corpus Christi, TX 78404 USA

**Keywords:** Validity, iClicker, Audience response system, Childhood obesity, Diabetes

## Abstract

**Objective:**

Healthy habits during childhood has been of prime importance. We aimed to gather baseline information about health habits from children in kindergarten and first grade (typically ages 5–7). Our objectives were to validate the questionnaire in assessing health habits, as well as the electronic audience response system, iClicker (MPS, Gordonsville, VA), in this age group.

**Results:**

The questionnaire completed by 75 kindergarteners and 66 first graders. For the first graders, questions involving healthy choices were answered correctly 78% of the time (range 8–94%) and had 84% agreement on repeat testing (range 64–93%). Questions on diabetes were answered correctly 79% of the time (range 65–94%) and had 85% agreement on repeat testing. Crohnbach’s alpha was calculated to determine the reliability of the questionnaire: on the revised kindergarten questionnaire, this ranged from 0.79 to 0.81 on Day 1 and 0.84–0.97 on Day 5; for the first graders, this ranged 0.79–0.81 on Day 1 and 0.84–0.97 on Day 5. Both kindergarteners and first graders answered the simplest of the basic knowledge questions correctly > 80% of the time, with acceptable test–retest agreement. Additionally, these children demonstrated acceptable understanding of the use of the iClicker classroom response system.

## Introduction

### Background

The prevalence of childhood obesity is rapidly increasing, reaching epidemic proportions [1]. Childhood obesity has been implicated in prediabetes/diabetes, dyslipidemia, hypertension, asthma, sleep apnea and a host of other long-term health implications [2–7]. The causes and contributors to childhood obesity are numerous, including hereditary factors, an increase in sedentary activities, and the proliferation of food and drink with high calorie content but otherwise poor nutritional component. Interventions aimed to curtail the childhood obesity epidemic have aimed at all of the causes, including behavioral modifications. Educational programs may provide a vital role, often starting in early childhood [8, 9]. Determination of baseline knowledge will allow educational programs to be optimized, and will allow for assessment of the efficacy of these programs. The “iConquer Program” (program developed by high school children to create healthy habits) is such an educational program aimed at improving knowledge of healthy eating and exercise habits, and basic diabetes facts in early grade school children (kindergarten and first grade, typically 5–7 years of age).

To our knowledge, no validated tool exists to assess the knowledge in this age group. Multiple-choice questionnaires remain a common method of obtaining responses from large groups; early school age children may not have sufficient experience with this format to be comfortable with it. Collecting and organizing responses from questionnaires may be accomplished by transcribing paper responses into computer spreadsheets and data analysis software. Portable computers and small touch-screen devices (smart phones, tablets, etc.) allow for direct entry of responses into the computer system, thus eliminating human errors in transcribing the data; this method is limited by cost and portability of some of the devices required. Electronic audience response systems can allow large groups of people to respond to individual questions at the same time, while accumulating the responses in a single computer file [10]. The user interface for these devices may be unfamiliar to some people, especially children, which may impact the accuracy of the responses. Therefore, both the questionnaire and the method of collecting responses need to be validated in order to be utilized effectively in this population.

## Main text

### Objective

We aimed to determine if baseline information about health habits from children in kindergarten and first grade (typically age 5–7 years) could be reliably gathered using a simple multiple-choice questionnaire and an electronic response system. An electronic response system was favored in an attempt to reduce costs and minimize the opportunities for assistance from teachers and other students. Of the electronic response systems commercially available, the iClicker classroom response system (MPS, Gordonsville, VA) was chosen for its simplicity and ease of use [10].

### Methods

#### Participants

The participants of this study were all students in kindergarten and first grade at an elementary school in Corpus Christi, TX. The questionnaire was completed prior to undergoing designated program, designated the iConquer Program, to educate children about healthy choices, including sleep, foods, and exercise. Permission for the educational program, including the questionnaire, was obtained from the Corpus Christi Independent School District and the local school administration.

#### Development and validation of the questionnaire

**Fig. 1:**
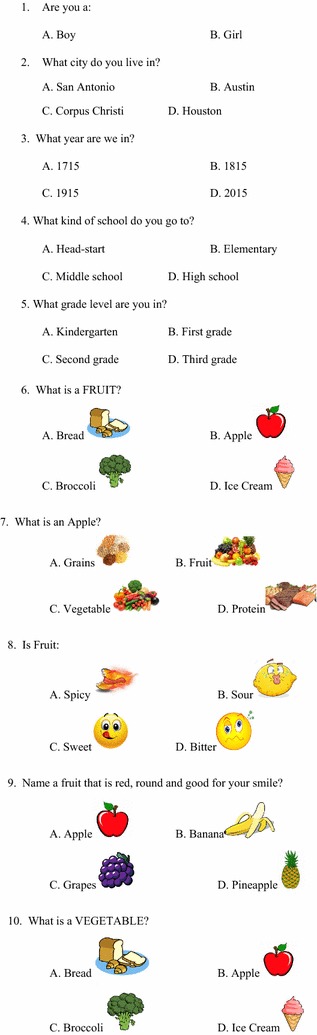
Original iConquer questionnaire

**Fig. 2:**
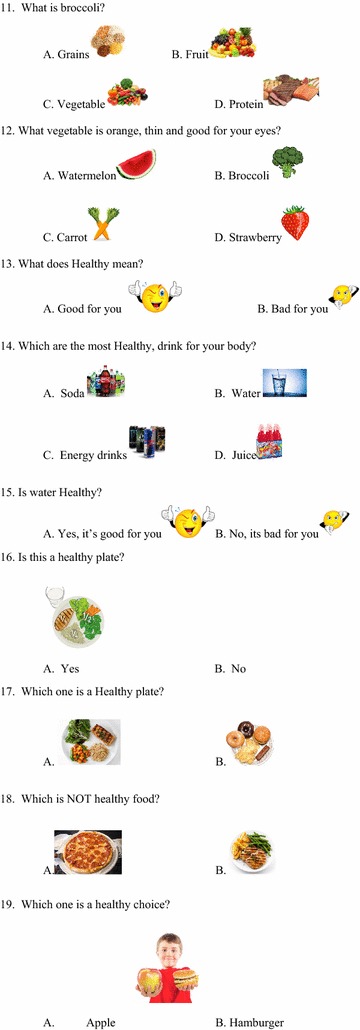
Original iConquer questionnaire (continued)

**Fig. 3:**
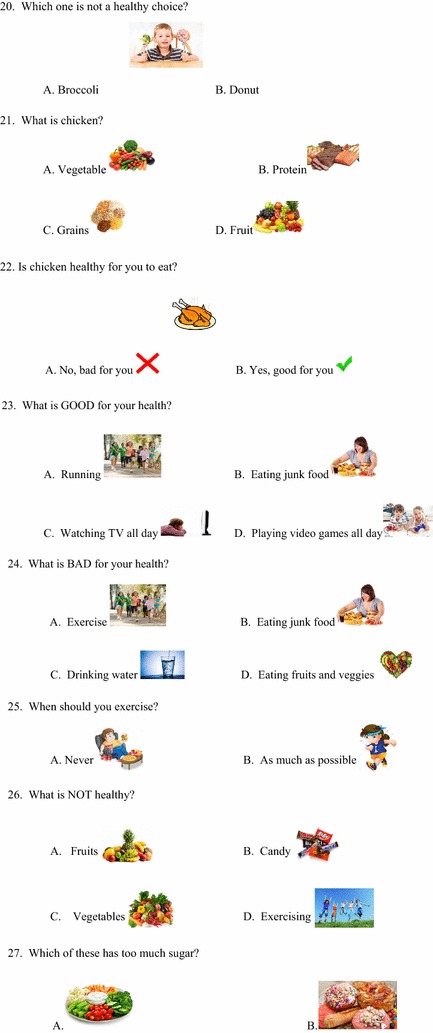
Original iConquer questionnaire (continued)

**Fig. 4:**
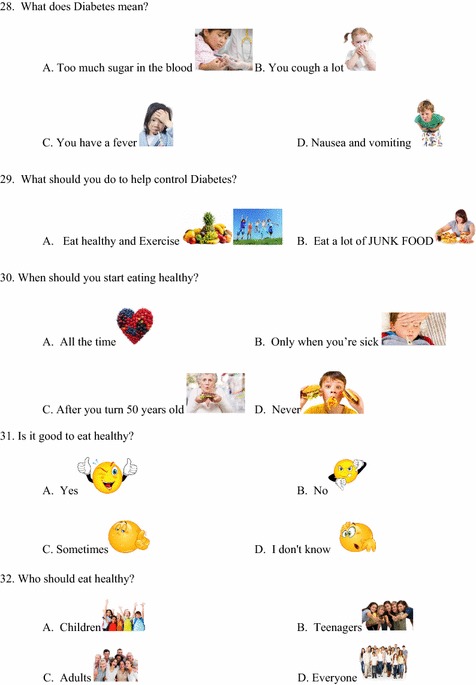
Original iConquer questionnaire (continued)

A questionnaire (Figs. 1, 2, 3, 4) was developed to assess baseline knowledge of kindergarteners and first graders at an elementary school in Corpus Christi, Texas. The questionnaire consisted of 32 multiple-choice items in total. The first 5 items queried non-specific, age-appropriate knowledge to allow for validation of the electronic answering system. The remaining 27 items covered healthy food choices, exercise and basic diabetes knowledge. The students completed the questionnaire using the iClicker classroom response system, then again in an identical manner 5 days later without specific intervention or education.

**Fig. 5:**
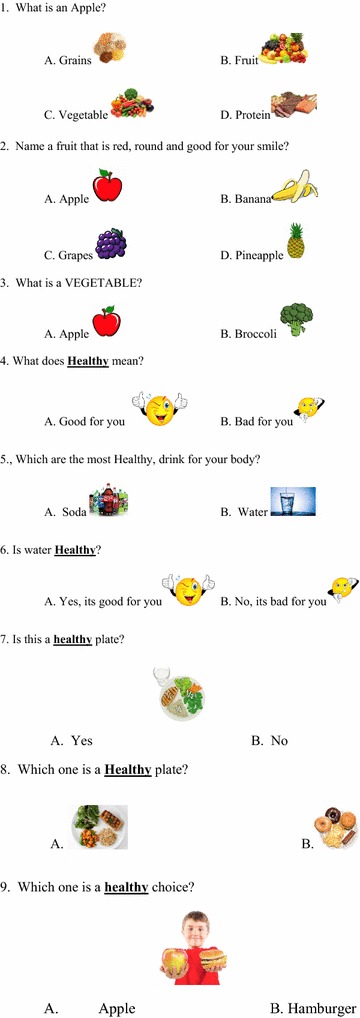
Revised iConquer questionnaire for kindergarten

**Fig. 6:**
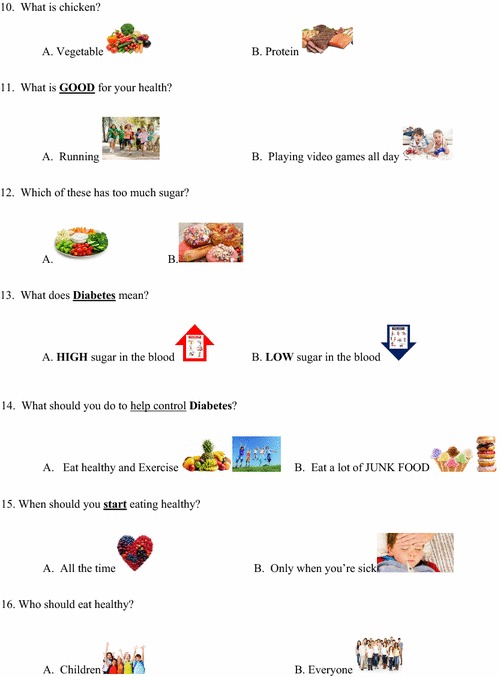
Revised iConquer questionnaire for kindergarten (continued)

The kindergarten questionnaire was then revised (Figs. 5, 6) to address systematic errors in the responses, felt to be related to the type of question and the length of the questionnaire. The initial 5 non-specific questions were eliminated; a total of 16 items were selected that showed adequate test–retest agreement and covered the desired subject matter. Additionally, the number of multiple-choice answers was decreased (from 4 to 2) in some of the questions. The revised questionnaires were then completed by the same kindergarten class, then repeated 5 days later without specific intervention or education.

#### Validation of the response system

Questionnaires were completed by the participants using the iClicker classroom response system (MPS, Gordonsville, VA), an electronic audience response system. As this system has not yet been validated in the age group of interest, the first 5 items of the questionnaire were designed to test the baseline validity of the response system (Figs. 1, 2, 3, 4). The students then completed the questionnaire in an identical manner 5 days later without specific intervention or education. These 5 questions were eliminated from the subsequent class-specific questionnaires.

#### Statistics

Analysis of response rates for individual questions were made via Fisher’s exact test (contingency analysis). A p value of < 0.05 was considered significant. Internal validity of the questionnaire for each group was calculated via Cronbach’s alpha.

Cronbach’s alpha provides a lower bound on reliability of a test. It has a maximum value of 1, and usually a minimum of 0, however, it can even be negative. It is also important to notice that when the number of items decrease, Cronbach’s alpha tend to decrease even without an actual decrease in internal consistency. A common rule of thumb is that a Cronbach’s alpha of at least 0.6 indicates acceptable reliability and 0.8 or higher indicates good reliability. Very high reliability is usually not desirable (greater than 0.95), as it may indicate redundancy on test items. The main goal in reliability is that the items must be internally consistent but with each item providing some individual contribution. Several analyses were performed to measure internal reliability of the instrument using Cronbach’s alpha as described at the results section below. Hypothesis tests for proportions were used to detect difference in correct response rates for some individual items, as it is shown at the next section. A significance level of 0.05 was used for hypothesis testing.

### Results

#### Participants

The questionnaire was at least partially completed by 75 kindergarteners and 66 first graders. Although individual demographics for each student are unavailable (other than gender), the following data are available for the Corpus Christi Independent School District: 59.3 economically disadvantaged, 5.5% limited English proficient (5.0% in bilingual/English as a second language education), 45.5% at-risk, 79% Hispanic, 13.8% White, 3.9% Black/African American, 0.2% American Indian/Alaskan Native, 1.8% Asian, and 0.6% identify as 2 or more ethnicities [11]. Of the students that responded to the gender question appropriately, 46% of the kindergarteners and 54% of first graders indicated that they were male.

#### Kindergarten

The response rate by kindergarteners decreased toward the end of the original questionnaire: there was no difference between the response rate for kindergarteners and first graders on question #1 (p = 0.23 for Day #1, p = 0.19 for Day #5), though the response rate from kindergarteners was significantly less on question #32 (p = 0.003 for Day #1, p = 0.005 for Day #5). Therefore, a shortened 16-item questionnaire was developed, with most of the items utilizing only 2 answer choices. The revised questionnaire was then administered and re-administered at the same school. Correct responses on the original questionnaire for Day 1 were 69.1% (SD 15.3%) and for Day 5 were 62.2% (SD 13%). On the revised questionnaire, correct responses for Day 1 were 74.6% (SD 8.8%) while for Day 5 were 71.8% with SD 8.9%.

For the original questionnaire, the Cronbach’s alpha coefficient ranged 0.80–0.83 (mean 0.82) for Day 1 and 0.86–0.88 (mean 0.87) on Day 5. For the revised questionnaire, the Cronbach’s alpha for Day 1 was 0.51–0.60 (mean 0.58) and for Day 5 was 0.80–0.82 (mean 0.82).

#### First grade

Questions involving healthy choices (24 questions) were answered correctly 78% of the time overall (range 8–94%) and had 84% agreement on repeat testing (range 64–93%); 6% of the questions were not answered. Questions on diabetes (3 questions) were answered correctly 79% of the time overall (range 65–94%) and had 85% agreement on repeat testing; 7% of the questions were not answered. The Cronbach’s alpha coefficient ranged 0.79–0.81 (mean 0.80) for the entire questionnaire on Day 1 and 0.84–0.97 (mean 0.85) on Day 5.

#### Validation of the response system

Results of the validation questions (first 5 questions) are shown in Table 1. Both kindergarteners and first graders answered the questions, “what city do you live in?” and “what grade level are you in?” correctly ≥ 88% in both instances, with ≥ 84% test–retest agreement. The question on gender generated acceptable test–retest agreement in both classes. First graders answered the questions, “what year are we in?” and “what kind of school do you go to?” correctly ≥ 70% of the time, with 75% agreement when retested; kindergarteners did not perform as well on these questions. For all 5 questions, kindergarteners averaged 74% correct answers and 75% agreement; first graders averaged 81% correct and 80% agreement.

**Table 1:** Responses to validation questions

Question	Kindergarten correct	Kindergarten test–retest agreement (%)	First grade correct	First grade test–retest agreement (%)
1. Are you a boy or a girl?	N/A	90	N/A	79
2. What city do you live in?	96%	89	89%	84
3. What year are we in?	85%	60	70%	75
4. What kind of school do you go to?	44%	52	79%	75
5. What grade level are you in?	88%	86	88%	87

## Discussion

This study has shown that early grade school children are able to utilize the iClicker electronic audience response system adequately to answer multiple-choice questions. Additionally, the responses to the multiple choice questions were consistent on retesting and appear to sufficiently assess the children’s knowledge of healthy habits and basic diabetes information.

Previous published reports studies have demonstrated effective use of the iClicker system (and other clickers) in older users [12, 13], often used to engage students in large classroom lecture environments [14, 15]. While online claims by vendors of audience response systems [16], and others [17], suggest that these systems benefit learning and student engagement, there is little data to validate the use of these systems in early grade school children [18]. We have shown here that both kindergarteners and first graders were effectively able to use the iClicker response system in answering the newly designed multiple-choice questionnaire.

The response rates on the questions from kindergarteners were significantly lower than those from the first graders, a trend that was magnified later in the questionnaire, suggesting that the length of the questionnaire (i.e., fatigue) played a role in non-responses, which is consistent with the children short attention span. Kindergarteners are often less familiar with the format of multiple choice tests [19], so changes were made in an attempt to put the focus on the content rather than the format. These changes included reducing the number of items, reducing the number of answer choices in the majority of the items, and eliminating many of the negative phrases in many of the question stems. The response rates to the shorter revised questionnaire were markedly improved. These strategies should be considered when developing similar questionnaires in the 5–6-year-old age group.

## Conclusions

The iConquer questionnaire reliably assesses the knowledge of 5–6-year-old children on healthy lifestyles and the basic understanding of diabetes. The iClicker audience response technology appears to be a valid tool for obtaining questionnaire responses from this age group. Similar questionnaires developed for kindergarteners should limit the number of questions and the number of answer choices.

## Limitations

Limitations of this study include the use of a single school as the subject pool. This design was purposeful in order to reduce variability and allow for test–retest comparisons; however, it limits the generalizability of the results. Even though there was no educational intervention done between any of the testing sessions, the same kindergarteners may have answered the same or similar questions up to four times, leading to possible improvement based on practice effect. This study does not address the socioeconomic status, race and ethnicity among the children, in addition to the cultural differences in the children in developing countries.

Future use of the iConquer Program should focus on 2 matters. First, the questionnaire and audience response system should be applied to a broader population of early grade school children to confirm generalizability. Second, the questionnaire should be given before and after an educational intervention to assess the effect of the teaching. Longitudinal studies may then assess the effect of that teaching on aspects of childhood health, such as obesity and diabetes.
